# Farmers’ beliefs and concerns about climate change, and their adaptation behavior to combat climate change in Saudi Arabia

**DOI:** 10.1371/journal.pone.0280838

**Published:** 2023-01-25

**Authors:** Muhammad I. Azeem, Bader Alhafi Alotaibi

**Affiliations:** Department of Agricultural Extension and Rural Society, College of Food and Agriculture Sciences, King Saud University, Riyadh, Saudi Arabia; Neijiang Normal University, CHINA

## Abstract

Climate change threatens the existence of humankind on the planet Earth. Owing to its arid climate and poor natural resources base, Saudi Arabia is particularly susceptible to the negative impact of ongoing climate change. Farmers’ understanding of this global phenomenon is extremely important as it may help determine their adaptation behavior. This study was designed to analyze farmers’ beliefs and concerns about climate change as well as their views about adaptation different obstacles. Data were collected from 80 randomly farmers of the Al-Ahsa region in Eastern Province using structured interviews. The findings revealed that farmers believed that climate change is mainly occurring due to anthropogenic activities. Drought, insects, crop diseases, and heat stress were their main concerns regarding adverse impacts of climate change. Lack of knowledge about adaptation practices, and poor government and financial support are perceived as the major obstacles to adaptation. The results of non-parametric analysis identified no significant differences in farmers’ climate change beliefs and concerns, and their views about obstacles to adaptation in relation to their demographic characteristics. Based on the findings, we suggest that capacity building programs should be undertaken by the government for enhancing the adaptive capacity of the farmers as well the provision of financial incentives wherever deemed necessary for promoting the adoption of sustainable agricultural practices and building a resilient national food system.

## 1. Introduction

Across the globe, climate change has emerged as a serious issue with significant implications for various domains of human life [1–3]. It poses a serious threat to the economies and societies of the world [4]. Owing to changes in the climate, global warming as well as a change in precipitation patterns is predicted. By the end of 2100, global average temperatures may rise by 1.4–5.8 degree Celsius [5]. Across different regions of the world, shifts in seasonal water availability throughout the year are also likely to be induced [6]. The frequency and intensity of extreme weather events like flooding and drought may also increase [7–9].

Aa a result of changes in the climate, global agricultural production systems and food security are threatened [3,10–13]. The productivity of both irrigated and rain-fed agriculture will be considerably affected. Majority of the world’s population is anticipated to experience the potential negative impacts of climate change. It is anticipated that in many regions, there will be a decrease in crop productivity [14–16]. Climate change is likely to enhance the impact of both biotic and abiotic stresses on agriculture [17]. According to the WHO projections, around 540–590 million people will be undernourished at a warming of 2 degree Celsius [18]. Climate-induced water scarcity may cause some parts of the world to lose their up to 6% of their national Gross Domestic Product (GDP) [19]. The most serious impacts will be in those regions that are already vulnerable to food insecurity and rural poverty [20].

Saudi Arabia is characterized by an arid climate [5,21]. In some parts, temperatures can be more than 50 degree Celsius [5]. Rainfall is extremely limited; long-term average precipitation is around 100 mm per annum. However, in Western parts of the Kingdom, rainfall can rise up to 500 mm per annum [5]. Intense and frequent precipitation events are rare [22]. Over the last 50 years, a 1.9 degree Celsius increase in average temperature was observed [23]. The rate of increase was faster (0.72 degrees C per decade) in the dry season as compared to the wet season (0.51 degrees C per decade) [24]. Several studies predict a 2–4 degree Celsius increase in the average temperature in the Kingdom by the end of 2100 due to ongoing climatic changes [25–27]. Although significant change in rainfall has not been observed during last 50 years [23], future precipitation projections however suggest a decrease in rainfall in many parts of Saudi Arabia [27,28]. The Kingdom lacks recurrent rivers and permanent water bodies. Saudi Arabia, along with other countries of the Gulf Cooperation Council (GCC) has been classified as water-scarce nations by the United Nations [29]. According to Water Resources Institute, 14 out of 33 countries that are most likely to be water-stressed in 2040 would be in the Middle East, and among them, Saudi Arabia has been ranked at 9^th^ position [30]. Water scarcity increases vulnerability of the region to the impacts of climate change [31].

The arid climate of the Kingdom makes it highly vulnerable to the potential negative impacts of climate change. Variations in temperature and rainfall severely affect food production. Several studies have reported significant impacts of climate change on agriculture [32–34]. Date palm production in the Kingdom is predicted to decline significantly owing to unfavorable climatic conditions [34]. A 3–5 degree Celsius rise in temperature would pose serious challenges for the agriculture and other economic sectors in the Kingdom [21,34]. Reduction in crop yields may range between 5–25% by just one-degree Celsius increase in temperature [23]. At 1 and 5 degree Celsius increase in temperature, irrigation water requirements of various crop would increase by 602 and 3,122 million Cusic meters, respectively [35]. Global warming could increase agricultural water demand around 5–15% to sustain current levels of agricultural production [26]. As 90% of agriculture in Saudi Arabia is irrigated, yields will be significantly reduced due to water scarcity [36]. About 70% of the annual water use is consumed by the agricultural sector [23]. A change in abundance and distribution of diurnal desert animals may also happen due to global warming [25].

A decrease in local food production will affect the national food security; food prices at the domestic level will be on rise, and it would also lead to higher food imports, increasing dependency of the Kingdom on other countries for its food security [37]. Therefore, the Kingdom is serious undertaking climate change adaptation and mitigation measures by employing various institutional options as well as facilitations of different stakeholders for combating this global problem [23]. Farmers are key stakeholders in this regard as they are not only directly affected by climate change, but their actions can also contribute towards climate change.

Globally, there is a consensus among different nations about the ongoing issue of climate change and its underlying causes. This paved the way toward the formulation of an international climate agreement in 2015 under the aegis of United Nations, commonly known as Paris Agreement. The agreement recognizes climate change as a global problem and therefore encourages all the countries to undertake collective actions to combat this issue by considerably reducing their carbon footprint [38]. At individual level, however, people may hold different beliefs about climate change and its causes. Farmers’ climate change beliefs refer to their acceptance and trust on various explanations of ongoing climate change. Such explanations include the ideas that climate change is either happening mainly due to human-induced activities or as a natural phenomenon or both. It also includes the ideas that there is insufficient evidence about climate change or climate change is not happening at all [39,40]. Understanding farmers’ beliefs about climate change is of considerable importance as they can influence their decisions to adopt certain appropriate climate change adaptation and mitigations measures. Moreover, it can help us predict their adaptation behavior and can assist in formulating effective extension and outreach initiatives for developing resilient food production systems [39–41]. Farmers’ concerns refer to the feelings of worry about the observed and potential impacts of climate change that can negatively affect agriculture and farm income. A huge body of literature is there that documents various observed and potential impacts associated with climatic changes [3,5,7,10,13,42–45]. The main impacts include drought, heat stress, flooding, increased incidence of crop diseases, insects and pests, higher weed infestations and invasive weeds, reduction in soil fertility etc. Farmers also confront various obstacles that can restrict their ability to effectively implement climate change adaptation and mitigation strategies. The exact nature and extent of theses may vary from region to region and farmer to farmer [42,46].

Across the globe, many studies attempted to analyze farmers’ views and responses to climate change in different countries [47–53]. In Saudi Arabia, majority of the research conducted about climate change and its likely impacts during the last decade followed a top-down approach and is attempted to predict the consequences of climate change on local scale. Only a few studies [41,54] directed targeted the farmers in the Northern region of Saudi Arabia. The present study was intended to fill these gaps and was carried out in the Eastern region that is a rather neglected part of the Kingdom in this regard. The study was carried out to achieve the following research objectives:

1. To identify farmers’ beliefs about climate change
2. To identify farmers’ concerns about impacts of climate change
3. To identify farmers’ views regarding various obstacles to adaptation
4. To determine differences in farmers’ beliefs, concerns and views about obstacles to adaptation due to demographic characteristics

## 2. Methodology

### 2.1. Description of the study area

**Fig 1:**
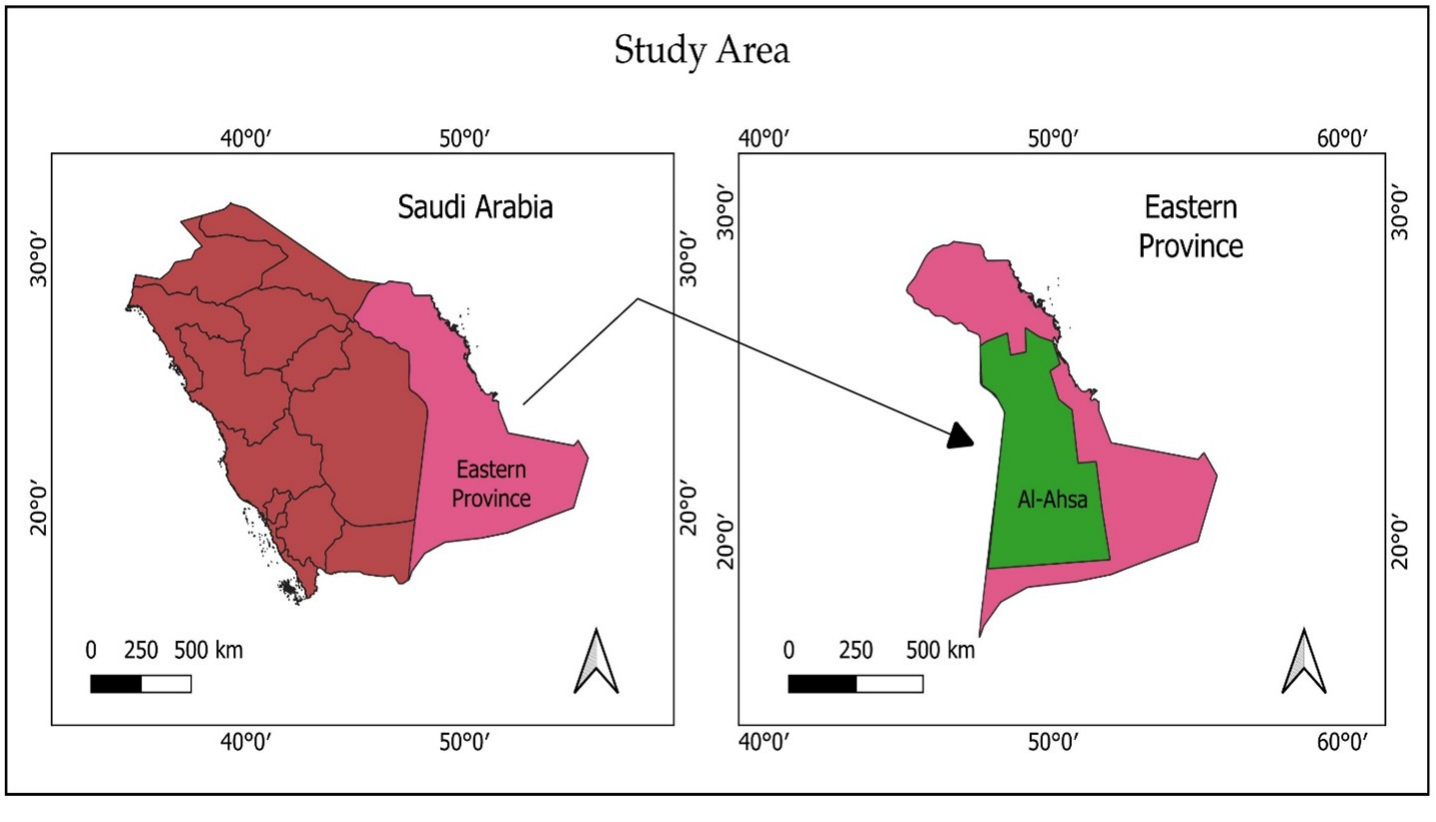
Map of the study area.

This study was conducted in the Al-Ahsa governorate of the Kingdom. It is part of the Eastern Province of the Kingdom, which comprises of 11 different governorates. In terms of area, Eastern Province is the largest region of the Kingdom and third most populous after the Riyadh and Makkah provinces. The total population of the Eastern Province is around 0.514 million; the population of Al-Ahsa governorate is about 0.2 million [55]. Al-Ahsa is the largest governorate of the Eastern Province. It has the world’s largest oases and is an important area for date palm production. It is a green governorate and contains fertile lands in the Eastern Province [56]. Fig 1 shows map of the study area.

### 2.2. Research design

This study was carried out using a cross-sectional survey design. The selection of Al-Ahsa governorate as the sampling area was purely made on the basis of its agricultural importance to the Eastern Province. Much of the agricultural activities in the Eastern Province are carried out in this governorate; the area has many date palm farms that also cultivate fruits, vegetables and other field crops in interspaces between date palm trees. Keeping in view the dates production in the area, the Ministry of Environment, Water and Agriculture (MEWA) established a modern date processing factory in the recent past. A group of researchers and experts in the Department of Agricultural Extension and Rural Society, King Saud University, Riyadh developed and validated the survey questionnaire before data collection. A list of farmers was obtained from the Directorate of Agriculture, Eastern Province (A working body of Ministry of Environment, Water and Agriculture, Saudi Arabia). The list provided details of about 191 farmers of Al-Ahsa region. Using simple random sampling, 80 farmers were selected from the list for data collection. Before data collection, farmers’ informed consent was verbally obtained. It was explained to them in a candid manner that their participation in the study is not compulsory and the collected data will only be used for academic purposes. Data were collected using structured interviews. Questions were asked using same wording and in the same ordering to all the respondents following a standardized pattern. The duration of each interview was between 30–40 minutes. The data collection process lasted for two months, from January to February 2022.

### 2.3. Research instrument

The survey questionnaire was divided into different parts to collect desired data. The first part contained questions related to demographic characteristics of the farmers. These included age, farming experience, farm size, level of education, nature of cultivation, extent of extension services received, farm ownership, cooperative agricultural membership, access to loans, and level of soil fertility in their farms. The second part comprised of the questions related to farmers’ beliefs about climate change and their sources of information regarding climate change. The beliefs of the farmers were measured on a five-point Likert scale (1 = Strongly Disagree; 2 = Disagree; 3 = Neutral; 4 = Agree; 5 = Strongly Agree). The question statements were adopted and modified accordingly based on a previous study conducted by Arbuckle et al. [40].

The third part of the instrument included questions related to farmers’ observation about changes in rainfall, temperature, and crop productivity over the last 15 years. This section also contained questions related to farmers’ concerns about potential problems due to climate change on their agricultural farms. There were eight Likert-type questions measured on a 4-point Likert scale (1 = Not Concerned; 2 = Slightly Concerned; 3 = Concerned; 4 = Very Concerned). The last part of the questionnaire included questions related to obstacles that farmers face in their adaptation efforts to climate change. There were eight questions measured on a 5-point Likert scale (1 = Strongly Disagree; 2 = Disagree; 3 = Neutral; 4 = Agree; 5 = Strongly Agree).

### 2.4. Data analysis

**Fig 2:**
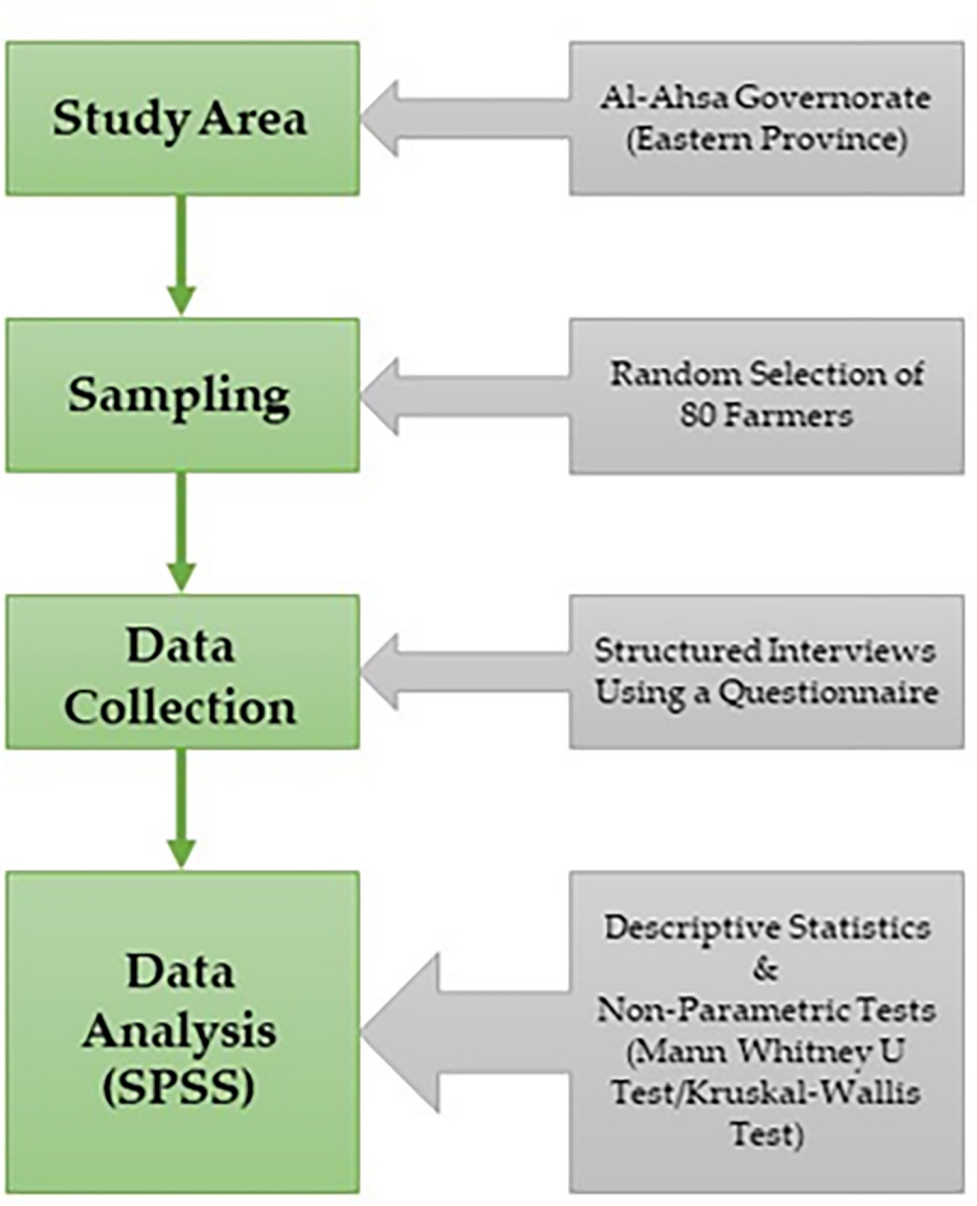
Methodological framework.

A range of both descriptive and inferential statistical tools were employed to analyze the collected data. The variable of annual farm income was deleted as about half of the farmers did not disclose their annual agricultural income. Farmers’ beliefs about climate change, their concerns about potential problems, and their views on adaptation obstacles were measured using a set of Likert scale questions. These variables were summed using related statements and three new variables were created based on the individual average scores of the farmers; the computed variables were treated as interval variables for running non-parametric analysis. To find differences in farmers’ beliefs regarding climate change, their concerns about impacts of climate change, and their views towards various adaptation obstacles due to demographic factors and some other selected variables, non-parametric tests were used. For the variables of membership of agricultural cooperatives, access to extension services, access to agricultural loans, and communication with other farmers about climate change, Mann Whitney U test (also known as Mann Whitney Wilcoxon test) was performed. On the other hand, for the variables of age, level of education, farming experience, farm size, farm ownership, soil fertility level, Kruskal-Wallis test (a non-parametric alternative of One-Way ANOVA) was used. Statistical Package for Social Science (SPSS 27.0v IBM) was used for the entire data analysis. Fig 2 provides an overall view of the methodological framework.

## 3. Results

### 3.1. Demographic characteristics of the farmers

Table 1 depicts the demographic characteristics of the farmers. About half of the farmers (51%) were above 50 years of age. Around 49% of the farmers were having at least bachelor or higher level of education; 35% had high school or below than it. About 49% had less than 20 years of farming experience; 51% of them had 20 years or more farming experience. The farm size of around 62% of the farmers was up to 4 hectares or below5; around 38% of the farmers had farms of more than 4 hectares in size. Nearly two-thirds of the farmers (65%) were the owners of their farms; 19% of them run their farms on rent whereas around 16% of them had shared farmland. Most of the farmers (71%) cultivated other crops in addition to fruits and vegetables on their lands. The perceived level of soil fertility at most of the farms (70%) was reported as average; only 16% reported high soil level of soil fertility at their farms. Majority of the farmers (87%) were not members of the agricultural cooperatives. Over half (55%) of the farmers were not having access to extension services. Around 76% of the respondents reported no access to agricultural loans. More than half (62%) were having communication with other farmers about climate change.

**Table 1:** Farmers’ demographic characteristics.

Variable	Frequency (n = 80)	Percent	Variable	Frequency (n = 80)	Percent
**Age (Years)**			**Farm ownership**		
20–40	23	28.7	Rented	15	18.8
41–50	16	20.0	Shared	13	16.3
51–60	25	31.3	Owner	52	65.0
Above 60	16	20.0	Nature of farming activities*		
**Level of education**			Vegetables	33	41.3
Less than High School	12	15.0	Fruits	32	40.0
High School	16	20.0	Other Crops	57	71.3
Diploma	13	16.3	**Soil fertility level**		
Bachelor	28	35.0	Low	11	13.8
Graduate School	11	13.8	Average	56	70.0
**Farming experience (Years)**			High	13	16.3
Less than 10	20	25.0	**Access to extension services**		
11–20	19	23.8	Yes	36	45.0
21–30	23	28.7	No	44	55.0
Above 30	18	22.5	**Access to agricultural loans**		
**Farm size (Hectares)**			Yes	19	23.8
Less than 1 ha	31	38.8	No	61	76.3
1–4 ha	19	23.8	**Communication with other farmers about climate change**		
Above 4 ha	30	37.5	Yes	50	62.5
**Membership of agricultural cooperatives**			No	30	37.5
Yes	10	12.5			
No	70	87.5			

*More than one answers were allowed; percentages of categories do not add up to 100.

### 3.2. Climate change beliefs of the farmers

Table 2 describes beliefs of the farmers about climate change. Over two-thirds (67%) of the farmers believed that climate change is occurring due to anthropogenic activities. Half (50%) of the farmers disagreed that climate change is happening due to natural changes. Around 41% of the respondents agreed that climate change is occurring due to both anthropogenic and natural changes. About 64% of the farmers disagreed that there is insufficient evidence about the occurrence of climate change; in other words, they were convinced that there is sufficient evidence about the occurrence of climate change. Majority of the farmers (84%) also expressed disagreement with the statement that there is no occurrence of climate change.

**Table 2:** Frequency distribution of climate change beliefs of the farmers.

Statement	Strongly Disagree (%)	Disagree (%)	Neutral (%)	Agree (%)	Strongly Agree (%)	Mean (n = 80)	Standard Deviation
Climate change is occurring due to anthropogenic activities	8.8	11.3	12.5	50.0	17.5	3.56	1.16
Climate change is occurring due to natural changes	11.3	38.8	17.5	22.5	10.0	2.81	1.20
Climate change is occurring both due to anthropogenic and natural changes	10.0	20.0	28.7	36.3	5.0	3.06	1.08
There is insufficient evidence about the occurrence of climate change	25.0	38.8	22.5	10.0	3.8	2.29	1.07
There is no occurrence of climate change	56.3	27.5	6.3	7.5	2.5	1.73	1.04

### 3.3. Farmers’ sources of information about climate change and their personal observations

Table 3 shows farmers’ sources of information about climate change and their personal observations of changes in rainfall, temperature, and crop productivity over the last 15 years. The findings revealed that farmers’ common sources of information about climate change were other peer farmers, internet, friends, mobile phone, and TV and radio. About 87% of the farmers reported that they observed a long-term decrease in rainfall over the last 15 years. In terms of temperature, a vast majority (92%) observed a long-term increase in temperature over the last 15 years. Over four-fifths opined an increase in temperature during last 20 years. Around 41% indicated a decrease in crop productivity during last 15 years; however, about 39% expressed that changes in crop productivity varied from crop to crop.

**Table 3:** Frequency distribution of the farmer’s sources of information and personal observations about climate change.

Variable	Frequency (n = 80)	Percent
Sources of information about climate change*		
Other farmers	67	83.8
Internet	67	83.8
Friends	64	80.0
Mobile	62	77.5
TV & radio	59	73.8
Newspapers	41	51.2
Agricultural extension	28	35.0
Cooperative agriculture	21	26.3
I do not have any sources	11	13.8
**Observing any long-term changes in rainfall over the last 15 years**		
Decrease in rainfall	70	87.5
Increase in rainfall	10	12.5
**Observing any long-term changes in temperature over the last 15 years**		
Decrease in temperature	6	7.5
Increase in temperature	74	92.5
**Observing changes in crop productivity over the last 15 years**		
I do not know	6	7.5
No change	4	5.0
Varies from crop to crop	31	38.8
Decrease	33	41.3
Increase	6	7.5
**Opinion about changes in temperature during last 20 years**		
I do not know	5	6.3
No change	3	3.8
Decrease	6	7.5
Increase	66	82.5

*More than one answers were allowed; percentages of categories do not add up to 100.

### 3.4. Farmers’ concerns about adverse impacts of climate change

Table 4 lists farmers’ concerns regarding adverse impacts of climate change at their farms. Most of the farmers (74%) were concerned about drought. Flooding was not a source of concern by the majority of farmers (72%). About 32% expressed that they were slightly concerned about weeds. Three-fourths of the respondents expressed their concerns about insect pressure and crop diseases. About 37% of the farmers reported that they were concerned about soil erosion. Heat stress on crops was also a source of concerns by most of the farmers (71%). Nearly half (47%) of the farmers stated that they were not concerned about saturated soils and ponded water at their farms.

**Table 4:** Frequency distribution of the farmers’ concerns about adverse impacts of climate change.

Potential Problem	Not Concerned (%)	Slightly Concerned (%)	Concerned (%)	Very Concerned (%)	Mean (n = 80)	Standard Deviation
Drought	13.8	12.5	56.3	17.5	2.77	0.90
Flooding	72.5	17.5	6.3	3.8	1.41	0.77
Weeds	31.3	32.5	28.7	7.5	2.12	0.94
Insect pressure	6.3	18.8	38.8	36.3	3.05	0.89
Crop diseases	6.3	20.0	38.8	35.0	3.03	0.90
Soil erosion	27.5	35.0	25.0	12.5	2.22	0.99
Heat stress on crops	10.0	18.8	45.0	26.3	2.88	0.91
Saturated soils and ponded water	47.5	20.0	20.0	12.5	1.98	1.09

### 3.5. Obstacles to adaptation for combating climate change

**Fig 3:**
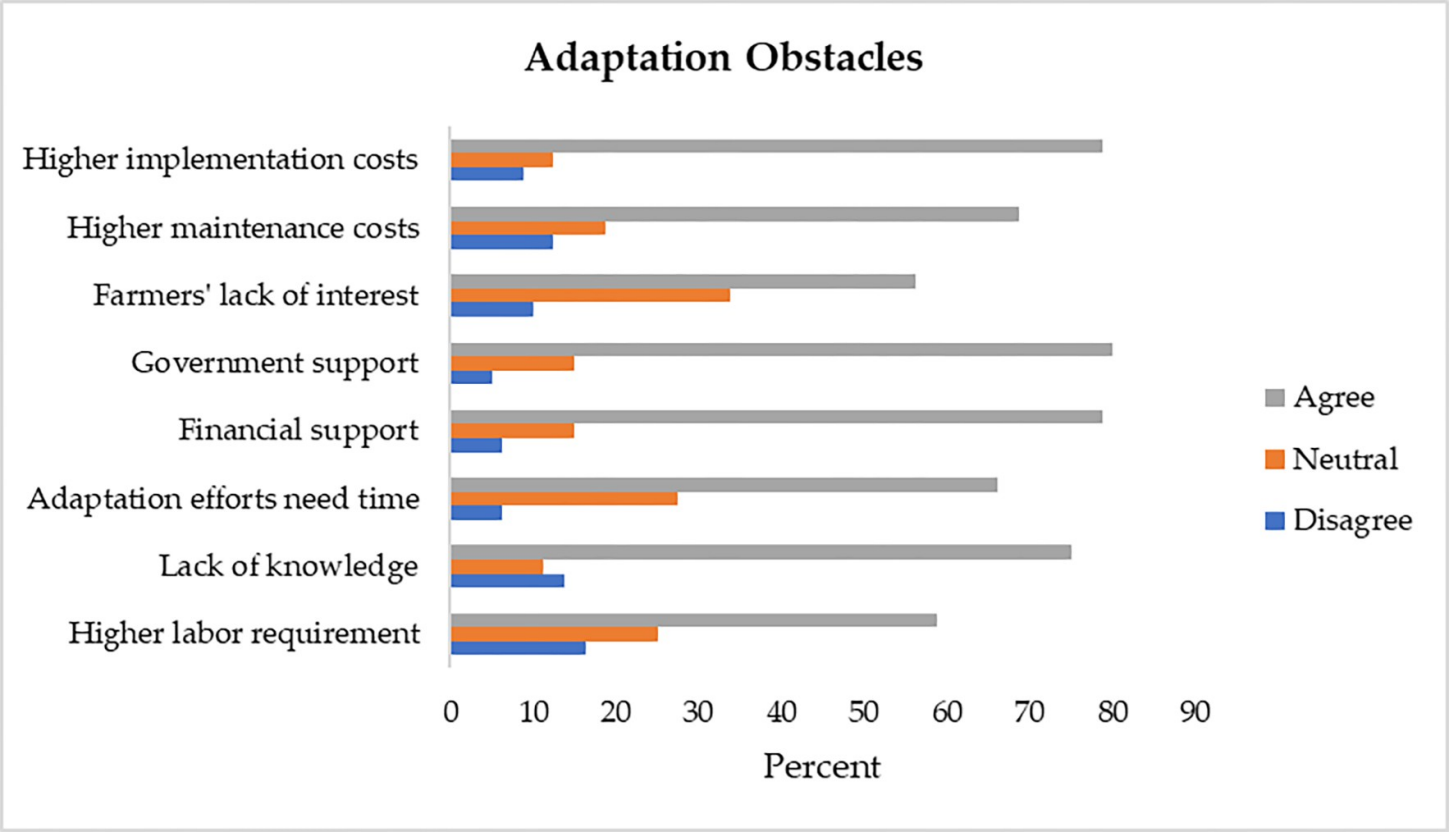
Farmers views about adaptation obstacles.

Table 5 shows farmers’ views about different obstacles to adaptation. Fig 3 presents the results after combing the five response categories to three. About 59% of the farmers had the opinion that adaptation requires more labor. Majority (75%) believed that they lacked proper knowledge about adaptation practices. Most of the farmers (66%) were also convinced that adaptation efforts need time for their implementation. Around 79% of the respondents believed that financial support is required for adaptation. Majority (80%) of the farmers were also convinced that adaptation also need government support. Although 56% expressed that only few farmers show interest in adaptation, but 39% were neutral about this issue. About 69% agreed that adaptation increases maintenance costs whereas 79% believed that adaptation implementation efforts are costly.

**Table 5:** Frequency distribution of the farmers’ view of obstacles to adaptation.

Obstacle	Strongly Disagree (%)	Disagree (%)	Neutral (%)	Agree (%)	Strongly Agree (%)	Mean (n = 80)	Standard Deviation
Adaptation requires more labor	6.3	10.0	25.0	47.5	11.2	3.47	1.03
Lack of knowledge about adaptation practices	3.7	10.0	11.3	65.0	10.0	3.68	0.92
Adaptation efforts need time	3.8	2.5	27.5	56.2	10.0	3.66	0.84
Adaptation efforts need financial support	2.5	3.7	15.0	33.8	45.0	4.15	0.98
Adaptation needs government support	2.5	2.5	15.0	35.0	45.0	4.17	0.95
Few farmers show interest to adapt	3.8	6.2	33.8	40.0	16.2	3.59	0.96
Adaptation increases the cost of maintenance	2.5	10.0	18.8	52.4	16.3	3.70	0.94
Adapting to climate change is costly to implement	2.5	6.3	12.5	51.2	27.5	3.95	0.94

### 3.6. Differences in farmers’ beliefs, concerns and their views regarding obstacles to adaptation according to demographic characteristics

Tables 6 and 7 show the results of non-parametric analysis run to identify differences in farmers’ beliefs about climate change, their concerns about the adverse impacts of climate change and their views about adaptation obstacles. The results of Mann Whitney U test revealed that there were no significant differences in farmers’ climate change beliefs, their concerns and views about adaptation obstacles due to their membership of agricultural cooperatives, access to extension services and access to agricultural loans. Significant differences in farmers beliefs about climate change were found due to their communication with other farmers about climate change. However, the determination of effect size showed a small effect (Cohen’s d = 0.22).

**Table 6:** Mann Whitney test for differences in beliefs and concerns about climate change and views about adaptation obstacles.

Variables	Beliefs about climate change			Concerns about potential problems			Views about adaptation obstacles		
	Mean Rank	Mann Whitney *U*	Sig 2-Tail	Mean Rank	Mann Whitney *U*	Sig 2-Tail	Mean Rank	Mann Whitney *U*	Sig 2-Tail
**Membership of agricultural cooperatives**									
Yes (n = 10)	39.65	341.5	0.90	39.45	339.50	0.87	29.65	241.50	0.11
No (n = 70)	40.62			40.65			42.05		
**Access to extension services**									
Yes (n = 36)	41.65	750.5	0.68	42.33	726.0	0.52	35.40	608.5	0.07
No (n = 44)	39.56			39.0			44.67		
**Access to agricultural loans**									
Yes (n = 19)	36.39	501.5	0.37	40.21	574.0	0.95	35.66	487.5	0.29
No (n = 61)	41.78			40.59			42.01		
**Communication with other farmers about climate change**									
Yes (n = 50)	36.46	548.0	0.04* Cohen’s d = 0.22	40.88	731.0	0.85	40.04	727.0	0.81
No (n = 30)	47.23			39.87			41.27		

*Significant at 0.05 level.

**Table 7:** Kruskal-Wallis test for differences in farmers’ beliefs, concerns, and views regarding adaptation obstacles.

Variables	Beliefs about climate change			Concerns about potential problems			Views about adaptation obstacles		
	Mean Rank	Chi-Square	*p*-Value	Mean Rank	Chi-Square	*p*-Value	Mean Rank	Chi-Square	*p*-Value
**Age (years)**									
20–40 (n = 23)	37.98	2.0	0.57	41.61	2.38	0.49	42.91	1.68	0.64
41–50 (n = 16)	42.49			43.53			37.06		
51–60 (n = 25)	37.40			34.80			43.50		
Above 60 (n = 16)	46.53			44.78			35.78		
**Education level**									
Below High School (n = 12)	30.54	6.25	0.18	41.79	2.07	0.72	42.83	2.55	0.63
High School (n = 16)	37.09			46.03			33.69		
Diploma (n = 13)	39.85			36.65			46.50		
Bachelor (n = 28)	48.48			41.05			39.59		
Graduate School (n = 11)	36.77			35.36			43.09		
**Farming experience (years)**									
Less than 10 (n = 20)	46.75	4.63	0.20	39.73	0.20	0.97	47.85	6.70	0.08
11–20 (n = 19)	39.16			40.03			38.03		
21–30 (n = 23)	43.24			42.33			44.48		
Above 30 (n = 18)	31.47			39.53			29.86		
**Farm size (hectares)**									
Less than 1 ha (n = 31)	47.52	4.91	0.08	40.87	0.01	0.99	40.50	0.40	0.81
1–4 ha (n = 19)	37.97			40.05			43.16		
Above 4 ha (n = 30)	34.85			40.40			38.82		
**Farm ownership**									
Rented (n = 15)	38.60	6.83	0.03*	44.10	0.45	0.79	30.63	3.41	0.18
Shared (n = 13)	55.73			40.27			41.31		
Owner (n = 52)	37.24			39.52			43.14		
**Soil fertility level**									
Low (n = 11)	54.91	5.88	0.053	45.23	1.27	0.53	35.05	1.22	0.54
Average (n = 56)	39.46			40.90			42.34		
High (n = 13)	32.77			34.77			37.19		

*Significant at 0.05 level.

The results of the Kruskal—Wallis test showed that there were no significant differences in farmers’ beliefs, concerns and views about adaptation obstacles due to their age, education level, farming experience, farm size, and soil fertility level. However, significant differences in farmers’ beliefs about climate change were observed due to farm ownership. The farmers who shared the farm were more likely to believe that climate change is occurring than those who owned the farm.

## 4. Discussion and implications

The current study attempted to explore farmers’ beliefs about climate change, their concerns about the potential impacts of climate change, and their views about different obstacles to adaptation in the Eastern Province of Saudi Arabia. Analysis of the farmers’ beliefs about climate change reveals that they believe that climate change is happening. They are also convinced that there is ample evidence to support the occurrence of climate change. Although, a small proportion of the farmers express that the ongoing climate change is either happening due to natural changes or both natural changes and anthropogenic factors, however, a majority holds the belief that climate change is happening mainly due to anthropogenic activities. These findings are consistent with several past studies [40,41,54,57] that reported that farmers are generally convinced about the occurrence of climate change. However, the current study differs on certain aspects from a previous study conducted by Alotaibi et al., [54] in the Jazan region located in the southwest of Saudi Arabia. The farmers of the Jazan region believed that climate change is occurring mainly due to natural changes. On the other hand, only a small number of the famers in the present study believe that natural changes are the main driver of climate change. This suggests that there could be regional differences in farmers’ beliefs about the causes of climate change that in turn may have an impact on their potential adaptation actions. Saudi Arabia is one of those counties that have ratified Paris Agreement and is actively involved in the implementation of United Nations’ Sustainable Development Goals (SDGs). Over the years, the government has launched media comp campaigns at the national level for creating awareness among the general public about the issue of climate change and its drivers. This might be the reason behind differences in beliefs of the farmers about climate change of the Eastern Province.

Farmers’ beliefs about climate change are also in line with the current scientific consensus regarding ongoing climate change, which also forms the basis of international agreement like Paris Climate Agreement. The Intergovernmental Panel on Climate Change (IPCC), a credible body of international scientists and researchers, maintains that ongoing climate change is a real threat to humankind and the main drivers behind this change are the anthropogenic Greenhouse Gas (GHG) emissions [42]. Major anthropogenic activities that substantially release GHGs to the atmosphere and are potentially disruptive to human beings include: fossil fuel burning; transport sector; industrial processes; and, agriculture, forestry and other land-use changes including deforestation [42,58,59].

Farmers’ concerns about the potential impacts of climate change are thought to originate from the risk associated with extreme weather events such as heat waves, drought, floods and cyclones [31,54,60]. The real nature and extent of the concerns may vary based on the level of exposure to such risks. Due to greater awareness about climate change worldwide, concern about the impacts of climate change have also increased [61]. The findings of the current study reveal that major concerns of the farmers are: drought, insects, crop diseases and heat stress. Personal observations of the farmers also suggest that they have witnessed a long-term increase in temperature, and a long-term decrease in rainfall and crop productivity over the last 15 years.

Agriculture is highly vulnerable to climatic variability. Changes in temperature and rainfall patterns will have a considerable impact on crop production globally. Climate change limits our capacity to maintain current levels of agricultural productivity [45]. There is a general consensus that over the last few decades average temperature in the Kingdom has risen. A recent study reported that a 1.9 degree Celsius increase in temperature was observed during the last 50 years. Moreover, It is projected that KSA will experience a 3–5 degree Celsius increase in temperature by the end of this century [25,27]. This would in turn significantly increase the irrigation water requirements of different crops grown in the Kingdom [35]. According to one study, there would be a 5–15% increase in water demand to maintain current production levels [26]. The projected increase in temperature could negatively affect the production of most of the crops by 5–25% [5,36]. A study argued that in the future, climate for date palm production might not be suitable in some parts of the Kingdom [34]. Saudi Arabia has been classified as a water-scarce nation along with other countries of the Gulf Cooperation Council (GCC) by the United Nations (UN) [29]. The Kingdom has very limited underground water resources; there are no permanent rivers. Unlike temperature, a significant change in rainfall was not observed during last 50 years [23]. Future precipitation projections however suggest a decrease in rainfall in many parts of Saudi Arabia [27,28]. This increases the vulnerability of the nation to adverse effects of climate change. The agriculture sector will be seriously affected in the Kingdom [23,31–34]. Drought is a significant driver of land degradation and its severity and spatial patterns will be greatly influenced by future greenhouse gas emissions [62–64].

Temperature and rainfall patterns also influence the population dynamics of insects and pests, crop diseases, weeds, and soil erosion. Global warming could exacerbate these threats by expand their geographical ranges and by altering their interactions with their hosts [65–70]. An increase in crop diseases would cause significant losses both in production and quality of the produce [71]. All these factors in combination pose a serious threat to the food security of many nations, especially developing countries and with poor natural resources base.

Similar findings were reported by Alotaibi et al. [54] in Jazan and Al-Mutairi et al [41] in Tabuk region in Saudi Arabia. However, the farmers of the current study were not concerned about flooding and saturated soils at their farms. It may be because of relatively row rainfall in the Eastern Province as compared to Jazan region. These findings are also consistent with the studies of Mase et al. [72] and Grimberg et al. [73] in USA and Orduño et al. [74] in Mexico, which reported that farmers of both USA and Mexico were concerned about drought, floods, insects, pests, and crop diseases.

The results of non-parametric tests show that demographic characteristics do not significantly influence farmers’ beliefs about climate change and their concerns about the potential impacts of climate change. This suggests that regardless of the demographic differences, farmers equally believe that climate change is happening and the human-induced changes are the main driver behind climate change. Moreover, their higher level of concerns suggests that they are equally exposed to various risks associated with the potential impacts of climate change. It may serve as a motivational factor for the farming community to undertake climate change adaptation and mitigation measures in order to reduce risks attached with the bad impacts of climate change. The farmers who hold the belief that climate change is happening and is a real threat to humankind are more likely to undertake certain actions to adapt to it and reverse its causes [40,54].

However, several past studies suggest that demographic, socioeconomic, and institutional characteristics possessed by the individuals can play a significant role in affecting their concerns about the impacts of climate change [75–78]. Some studies reported significant differences in farmers’ climate change beliefs due to their membership of agricultural cooperatives, access to loans [54], access to extension services [54,79–81], soil fertility [54,79,80], age [54,82], and farm size [54,81,83]. Ado et al. [84] however found no significant differences in farmers’ beliefs due to membership of agricultural cooperatives. Significant differences in farmers concerns were also found in some studies due to their access to loans [54,79,85]. Heath and Gifford, [86] reported a positive relationship between level of education and concerns. However, Kellstedt et al, [87] found no relationship between these two variables. Several studies also report negative or no relationship of the age with the level of concerns [88–91].

Climate change adaptation and mitigation are two different processes: adaptation refers to adjusting to climate change impacts in order to prevent or reduce the risks and damages associated with the impacts of climate change; mitigation is the reduction in GHG emissions in order to prevent or slow down the process of climate change [42]. While adapting to climate change, farmers may face a number of different obstacles of varying nature and intensity that can undermine their adaptation efforts and impede the overall adaptation process. The study findings show that higher labor requirements, lack of interest and knowledge about adaptation practices, poor financial and government support are the major obstacles that affect farmers’ adaptation efforts in the Eastern Province. Several previous studies conducted in different countries identified similar problems faced by the farming community at large [92–96].

Agriculture is a labor-intensive profession [97]. Much of the farm work in the Kingdom is carried out by the foreign workers from Bangladesh, India and Pakistan. Over the last few years, the cost of living has significantly increased due to governmental measures for the expatriates. It makes difficult for the local farm owners to procure cheap labor. The resident workers also show less interest for such remote jobs where wages are relatively low and there are also limited opportunities to supplement earning using some other paid part-time arrangements. Therefore, the issue of agricultural labor workforce may persist until the government decides to intervene by relaxing the working environment for the agricultural labor.

Lack of interest to adapt and poor knowledge about different adaptation practices might be attributed to weak extension and advisory setup a considerable size of the farmers does not have access to extension services. This is also evident in farmers sources of information about climate change; extension is not a common source of information about climate change by majority of the farmers. This suggests clear gaps in extension system that need to be corrected. Substantial investments in capacity building are required. Farmers should be educated about sustainable agricultural practices and climate-smart agriculture to promote the adoption of such practices. The sustainable management of agroecosystems depends on sound knowledge and technical skills about different sustainable agricultural practices. Diverse and adaptive knowledge base is essential due to changing nature of agriculture. A set of agricultural practices have been developed as a result of decades of research. Adoption of these practices would not only increase the efficient use of natural resources, but it will also mitigate the negative impact of agriculture on the environment. Capacity building programs will enhance the adaptative capacity of the farming community as well as various other stakeholders of the value chain [42,98–103].

Farmers also indicate that they need financial support as it is costly to implement different adaptation measures. Additionally, it also increases maintenance costs. The findings suggest that most of the farmers have small farms (below 4 hectares) and also do not have access to agricultural loans. The provision of agricultural subsidies and interest-free loans may prove helpful in solving their financial problems and might promote the process of adaptation. However, economic incentives can promote the adoption in the short run, but a change in farmers’ behavior by cultivating a sense of environmental stewardship is more beneficial for the long-term use and retention of such practices [104,105]. Besides financial support, farmers also need government support in several other affairs. Such support can come in the form of conducive regulatory frameworks at national and local levels that aim to facilitate the adaptation activities. The government should formulate pro-environmental regulatory frameworks that are also economically feasible and socially just. Although, the Ministry of Environment, Water and Agriculture (MEWA) has outlined its ambition in the national strategy for agriculture [106] to implement sustainable practices in the Kingdom, but concrete measures are needed to materialize the ambitious plan. In order to adopt a multisectoral adaptation strategy for promoting synergy between various departments, the government can create and strengthen cooperation and collaboration between interrelated and interdependent institutions. Cross-ministerial structures and multi-sector planning that aims to promote greater policy coherence can prove more effective for climate change adaptation [107,108]. Moreover, the government should focus on the creation of markets for sustainable products to encourage the farmers to adaptation in the long-run.

The findings also reveal that demographic characteristics do not significantly affect farmers’ views about different adaptation obstacles. It suggests that most of the farming community in the study area is facing same problems that need to be addressed by the concerned institutions.

## 5. Conclusion

The current study explores farmers’ beliefs about climate change, their concerns about the potential impacts of climate change, and their perspective regarding different adaptation obstacles. In terms of beliefs, farmers seem convinced about the occurrence of climate change and think that its major drivers are human-induced actions. Drought, insects, crop diseases, and heat stress on crops are their main concerns that increase their risks associated with the potential adverse impacts of climate change. In order to minimize these risks and vulnerabilities, farmers need to adopt a set of climate-smart and sustainable agricultural practices.

However, their lack of interest and poor knowledge about different adaptation practices can reduce their capacity to adapt to climate change impacts. These hurdles can be overcome by the government by investing in agricultural extension for improving farmers’ access to quality extension services that ensure their capacity development. Alotaibi et al, [54] have highlighted the weaknesses of public extension system in Saudi Arabia. Extension personnel need to be properly trained about the latest sustainable agricultural practices through in-service training programs in order to upgrade their knowledge and skills and to make this institution effective and relevant [109–112]. Financial constraints can be addressed by providing farmers with targeted agricultural subsidies and interest-free loans. Moreover, conducive regulatory frameworks and creation of markets for the farmers could ensure long-term adaptation by the farmers. Adoption of sustainable agricultural practices by the farming community will help the kingdom to implement Paris Agreement as well as achieve several Sustainable Development Goals (SDGs) related to poverty, food security and ecosystem protection.

### 5.1 Limitations of the study

The questionnaire was not pilot tested due to lack of time and money. Secondly, the present study only selected farmers from the Al-Ahsa region alone. Eastern province consists of 11 different directorates; future studies could select more than one directorate in order to better represent the region. Thirdly, in order to gain actual insights about farmers adaptation to climate change, future researchers may record various practices used by the farmers at their farms. This approach would be more practical in nature and would help us to analyze the actual situation in the field.
